# Exploring Filipino Medical Students’ Attitudes and Perceptions of Artificial Intelligence in Medical Education: A Mixed-Methods Study

**DOI:** 10.12688/mep.20590.2

**Published:** 2025-04-01

**Authors:** Robbi Miguel G. Falcon, Renne Margaret U. Alcazar, Hannah G. Babaran, Beatrice Dominique B. Caragay, Cheenie Ann A. Corpuz, Maegan Victoria S. Kho, Aleisha Claire N. Perez, Iris Thiele C. Isip-Tan

**Affiliations:** 1 College of Medicine, University of the Philippines Manila, Manila, Metro Manila, 1006, Philippines; 2 Department of Medical Informatics Unit, College of Medicine, University of the Philippines Manila, Manila, Metro Manila, Philippines; 3 Division of Endocrinology, Diabetes and Metabolism, Department of Medicine, Philippine General Hospital, University of the Philippines Manila, Manila, Metro Manila, 1006, Philippines

**Keywords:** artificial intelligence, medical education, chatbots, ChatGPT

## Abstract

Artificial intelligence (AI) has many implications on the practice of medicine, especially for current medical students who have to consider the impact of AI on the information available to patients and ethical aspects of rendering healthcare as a whole. With the fast pace of development in AI in healthcare, medical educators struggle to incorporate AI in the curriculum. The current generation of medical students will likely be the first to use AI tools in their practice, hence this study aims to investigate the current perceptions of medical students on the role of AI in medical education and the practice of medicine using a mixed methods parallel convergent design. The findings revealed that medical students had a baseline understanding of AI and its role in medicine, but required further training for its practical and ethical use. Moreover, the impact of AI in terms of their future practice in medicine (i.e., choice of specialization, doctor-patient relationship) was evident and must be considered by educators in order to promote responsible use of AI by physicians-in-training. In conclusion, findings from this study helped identify key areas of focus for integration into the medical curriculum related to the ethical use of AI in both education and clinical practice.

## Introduction

The Philippine medical curriculum traditionally followed the Flexnerian curriculum which focused on the science of medicine. This model draws much influence from Western methods of instruction, which are discipline-based and teacher-centered. The learning model has since evolved to incorporate community-based education, problem-based education, organ systems integrated models, and outcome-based education ( Sana *et al*., 2022). These changes occurred due to the limitations of the traditional system and the clamor for considering social accountability as an outcome of medical education ( Punzalan *et al*., 2023).

Filipino medical students are expected to be able to address pressing problems in the Philippine healthcare system and demonstrate the learning outcomes set by the Commission on Higher Education Memorandum Order No. 18 Series of 2016 ( CHED, 2016; Dioquino-Dimacali, 2017). In this memorandum order, medical informatics, but not artificial intelligence, is identified as required curriculum content. Medical students must also be able to maintain and contribute to the existing institutions for health by creating new knowledge and technology ( Baticulon *et al*. 2021; Sana *et al*., 2015; Sana *et al*., 2022). However, without adequately equipping students with the baseline knowledge and understanding of the fundamental applications of these tools, inequities which persist in relation to AI and the widespread digital divide in relation to socioeconomic disparities and barriers may further reinforce problems which already exist within the Philippine healthcare and medical education systems ( Alibudbud, 2024). The imminent shift to digital health ( Bajwa *et al*., 2021) necessitates medical schools to redefine key learning competencies to include the role of AI in the current teaching and learning paradigm, given its emerging role as a tool for students, healthcare professionals, and even patients alike ( Alowais *et al*., 2023; Ongkeko, 2024). Moreover, it is apparent that the Philippines has shown some level of receptiveness towards the use of AI in various educational sectors, but its role in medical education and training in other specialized disciplines has yet to be fully uncovered with the apparent limitations of the tool ( Giray *et al*., 2024). Of note are aspects of AI observed to have major implications on the perceptions of medical students such as the trust and rapport-building within the doctor-patient relationship, breach of professional confidentiality, and the impact on the humanistic aspect of medicine ( Gordon *et al*., 2024; Jackson *et al*., 2024).

The continued development of the practices in medicine and the medical educational system is often recognized in relation to the emergence of the digitalization of society, with the rise of modern technology and artificial intelligence (AI). These platforms have enabled the automation of various processes, allowing for greater efficiency in accomplishing tasks and prioritization of resources for more pertinent issues ( Al Kuwaiti *et al*., 2023). Moreover, these technologies have also opened several opportunities for improvements in the quality of doctor-patient interactions. By providing an avenue for patients to actively retrieve high-quality information about healthcare, previous studies have reported a drastic improvement in the proposed shift from a physician-centric to a patient-centric approach in rendering healthcare. However, with the advent of new technologies come some challenges that must be recognized and addressed to sufficiently integrate these tools into the practice of medicine. Previous investigations have reported the importance of comprehensively exploring the possibility of integrating competencies related to the role of AI in medical education ( Moldt *et al*., 2023). In professional practice, this is especially relevant in the context of medico-legal and ethical dilemma decision-making, diagnostics and therapeutic advancements, and health information. In the context of the healthcare profession's educational scheme, several studies have recognized the emerging role of AI in student interaction, personalizing learning didactics, and self-evaluations ( Shankar, 2022).

The emerging role of AI in the medical curriculum of aspiring physicians and the quality of healthcare being rendered by healthcare practitioners has yet to be explored in the Philippine setting. Although promising in improving the efficiency and ease of access to information for patients and doctors alike, problems such as the undermining of doctor-patient interactions, rigidity in history-taking and provision of care, and potential harm to patients in emergency situations due to overreliance on AI. In order to better equip future physicians with the tools necessary to effectively use and address concerns related to the use of AI in medicine, further studies are necessary to provide a proper basis for the inclusion of AI in medical education by elaborating on the role and impact of AI on the various facets of medicine. To achieve this goal, the current study aims to provide an overview of the current attitudes and perceptions of Filipino medical students enrolled in a medical informatics course in the College of Medicine, University of the Philippines Manila with regard to AI technologies to determine focal points for future implementation in the medical curriculum. The specific objectives of the study would consist of the following: (1) to understand how AI technologies work and how they affect the understanding of medical students in the context of healthcare provision and the future of medicine; (2) to determine the attitudes of medical students about the use of AI in the medical curriculum and practice of medicine.

## Methods

### Study design and participants

A mixed methods approach with a convergent parallel design was employed, adhering to the CONFERD-HP: recommendations for reporting COmpeteNcyFramEwoRk Development in health professions ( Batt *et al*., 2023). Twenty second-year medical students enrolled in the 2024 cohort of the UP College of Medicine elective course (Medical Informatics 220: Introduction to Medical Informatics) were invited to participate. The students were in the pre-clinical stage of their medical education, primarily focusing on foundational sciences with limited clinical exposure. Participants answered a questionnaire and took part in a focus group discussion regarding artificial intelligence in medical education and its impact on their future practice. Informed consent was obtained. Sample size estimation for saturation and data adequacy was not evaluated since the present study serves as the first pilot investigation to the perceptions and attitudes of Filipino medical students towards AI in medicine ( Hennink & Kaiser, 2022; Vasileiou *et al*., 2018).

### Ethical considerations

The study received ethical clearance from the University of the Philippines Manila Research Ethics Board (UPMREB 2024-0345-EX) and adhered to the WMA Declaration of Helsinki – Ethical Principles for Medical Research Involving Human Participants ( World Medical Association, 2024). Participation was voluntary, and students provided their written informed consent and received no compensation. Each study participant was adequately informed of the aims, methods, sources of funding, any possible conflicts of interest, institutional affiliations of the researcher, the anticipated benefits and potential risks of the study and the discomfort it may entail, post-study provisions and any other relevant aspects of the study. Participants were informed of the right to refuse to participate in the study or to withdraw consent to participate at any time without reprisal. All responses and data were anonymized and confidentiality was assured.

### Quantitative approach - survey

The questionnaire (Appendix A) had 24 questions, divided into five sections. Each section had a mix of five-point Likert scales and multiple-choice questions.

The questionnaire was divided into five subsections, with items including five-point Likert scales and validated multiple-choice questions based on previous surveys done on the topic of AI in medicine ( Civaner *et al*., 2022; Fischer *et al*., 2017; Moldt *et al*., 2023; Oh *et al*., 2019; Park *et al*., 2019; Wood *et al*., 2021). The first subsection from Civaner *et al*. (2022) and Moldt *et al*. (2023) focused on gathering demographic data including age, gender, and general attitudes toward technology and AI. Attitudes toward technology use were assessed using a shortened version of a validated scale for assessing individual differences in the willingness of technology (technology commitment) ( Fischer *et al*., 2017). The second subsection assessed the actual educational needs of medical students based on a previous survey used by Oh *et al*. (2019). This included questions related to the specific learning competencies and course content expected to be achieved in a module tailored to cover the role of AI in medicine, the openness of the student to implementation and future discussions of AI integration in relation to the practice of medicine (i.e., residency training, specialization) from Civaner *et al*. (2022), and the predicted impact of AI on medical education as a whole. The third subsection tackled the role of AI on the physician and their duties and responsibilities from a survey conducted by Wood *et al*. (2021). This included questions on both positive and negative predicted impacts of AI in the practice of medicine. The fourth subsection covered the patient’s perspective and how AI will shape the doctor-patient relationship by Civaner *et al*. (2022) and Oh *et al*. (2019). The final subsection covered ethical dilemmas and concerns related to the rational use of AI by Wood *et al*. (2021) and Moldt *et al*. (2023). This included questions on liability, ethical decision-making, and possible issues that may arise between the patient and doctor with the growing use of AI. The validity of the combined survey was evaluated using Oducado’s survey instrument validation rating scale (2020), which assessed (1) the subject, (2) clarity and ambiguity, (3) stability, (4) pull, (5) variation, (6) consistency, (7) inclusivity and (8) evaluability by external criteria.

Means, standard deviations, sum values, frequencies, and percentages of the relevant items were obtained using GraphPad Prism (version 8).

### Qualitative approach - focus group discussion

The participants were divided into two focus groups, each with 10 medical students, one facilitator, and one scribe for documentation. The focus group discussions ran in parallel, simultaneously. There were ten questions for discussion, divided into two sets: general perceptions of AI use and use of chatbots in healthcare. The first set of questions anchors the scope of the study and provides a baseline understanding of AI and its role in medicine. The second set of questions provides a theoretical scenario-based analysis of chatbots, to understand AI’s functional purpose and ease-of-use in medicine. Questions presenting ethical dilemmas were also included. The questions for the focus group discussion were based on a previous study by Moldt *et al*. (2023), which utilized an identical approach, with changes to the scope of the questions covering AI as a whole rather than just solely focusing on chatbots ( Ranganathan & Caduff, 2023).

### Integration

Findings from the quantitative survey and qualitative focus group discussion were integrated by connecting recurring ideas obtained, following Guetterman (2017). This involved generation of meta-inferences from the datasets and transcripts and legitimation of findings based on previous studies.

## Results

The participants of the study were composed of 20 medical students from the Medinfo 220: Introduction to Medical Informatics elective course in the College of Medicine, University of the Philippines Manila. Majority of the participants were male, and were aged 21 to 28 years old (μ = 23.1, SD: 1.6) (Extended Data 3).

### Survey

Majority of the participants were male (n = 11), and were aged 21 to 28 years old (Mean 23.1 ± 1.6 y).

### Baseline awareness

All participants had baseline knowledge of AI and the majority had an awareness of its emerging role in medical education and the practice of medicine (Extended Data 2). In terms of information sources, all students had received training through an elective course (Extended Data 2). Majority derived their information about AI from social media platforms ( *n* = 16), mentors ( *n* = 15), and online articles or news stories ( *n* = 14) (Extended Data 2).

### Educational Needs Assessment

**Figure 1.**
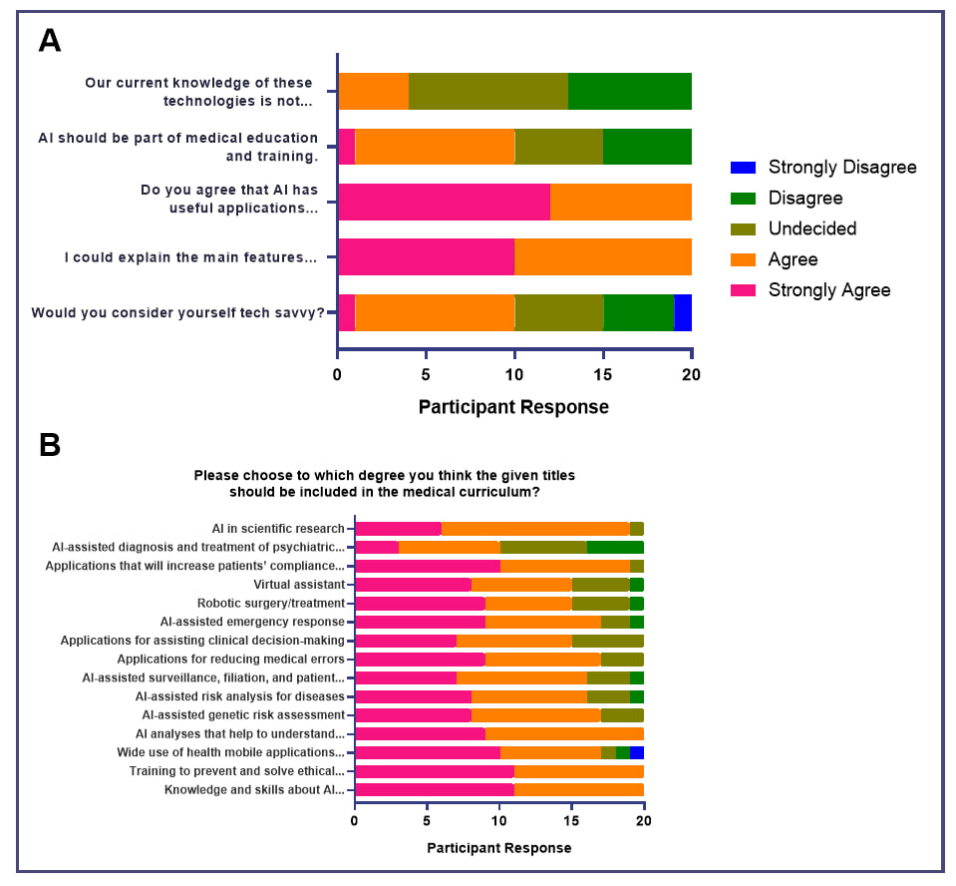
Attitudes of medical students toward AI in medical education (Educational Needs Assessment).

The majority agreed that our level of knowledge acquisition on AI was insufficient in allowing students to assess their benefits or risks to the health sector (45%, *n* = 9) and agreed (50%, *n* = 10) or strongly agreed (50%, *n* = 10) to the inclusion of AI-related topics in the medical curriculum ( Figure 1). Majority had either strongly agreed (60%, *n* = 12) or agreed (40%, *n* = 8) that AI had useful applications in the medical field. However, most were undecided in terms of being able to sufficiently explain the main features of AI and its applications (45%, *n* = 9), but affirmed their technological capacity (45%, *n* = 9) ( Figure 1).

When asked to which degree should certain AI-related topics should be included in the medical curriculum, all participants either strongly agreed or agreed (100%, n=20) to “knowledge and skills about AI applications”, “training to prevent and solve ethical problems that may arise with AI applications”, “AI analyses that help to understand and predict health phenomena”, “AI-assisted risk analysis for diseases”, “AI-assisted surveillance, filiation, and patient isolation in epidemics”, “applications for assisting clinical decision-making”. A few participants either strongly disagreed or disagreed with topics such as “wide use of health mobile applications in preventive healthcare” (10%, n=2), “applications for reducing medical errors” (10%, n=2), “robotic surgery or treatment” (5%, n=1), “virtual assistant” (5%, n=1), and “AI in scientific research” (5%, n=1). These suggest that while the majority of medical students are interested in learning about the different applications of AI, there may still be a debate whether or not the use of AI in scientific research and direct patient care such as disease prevention, surgery, or virtual assistance should be included in the curriculum ( Figure 1B).

### Impact on career

Generally, the majority believed that AI would affect some (30%, *n* = 6) to several (40%, *n* = 8) aspects of medicine (Appendix, Supplementary Table 1). In particular, the majority identified fields related to Radiology being affected the earliest and greatest by the introduction of AI (70%, *n* = 14). For interest in training and/or professional development topics, the majority were amenable to discussions of AI in both patient care and teaching (50%, *n*=10) (Extended Data 5). On the impact of AI on the students’ enthusiasm for residency or specialty training, the majority expressed that the early arrival of AI did not impact their enthusiasm or interest in their specialization of choice (55%, *n* = 11) (Extended Data 5).

### Impact on career

**Figure 2.**
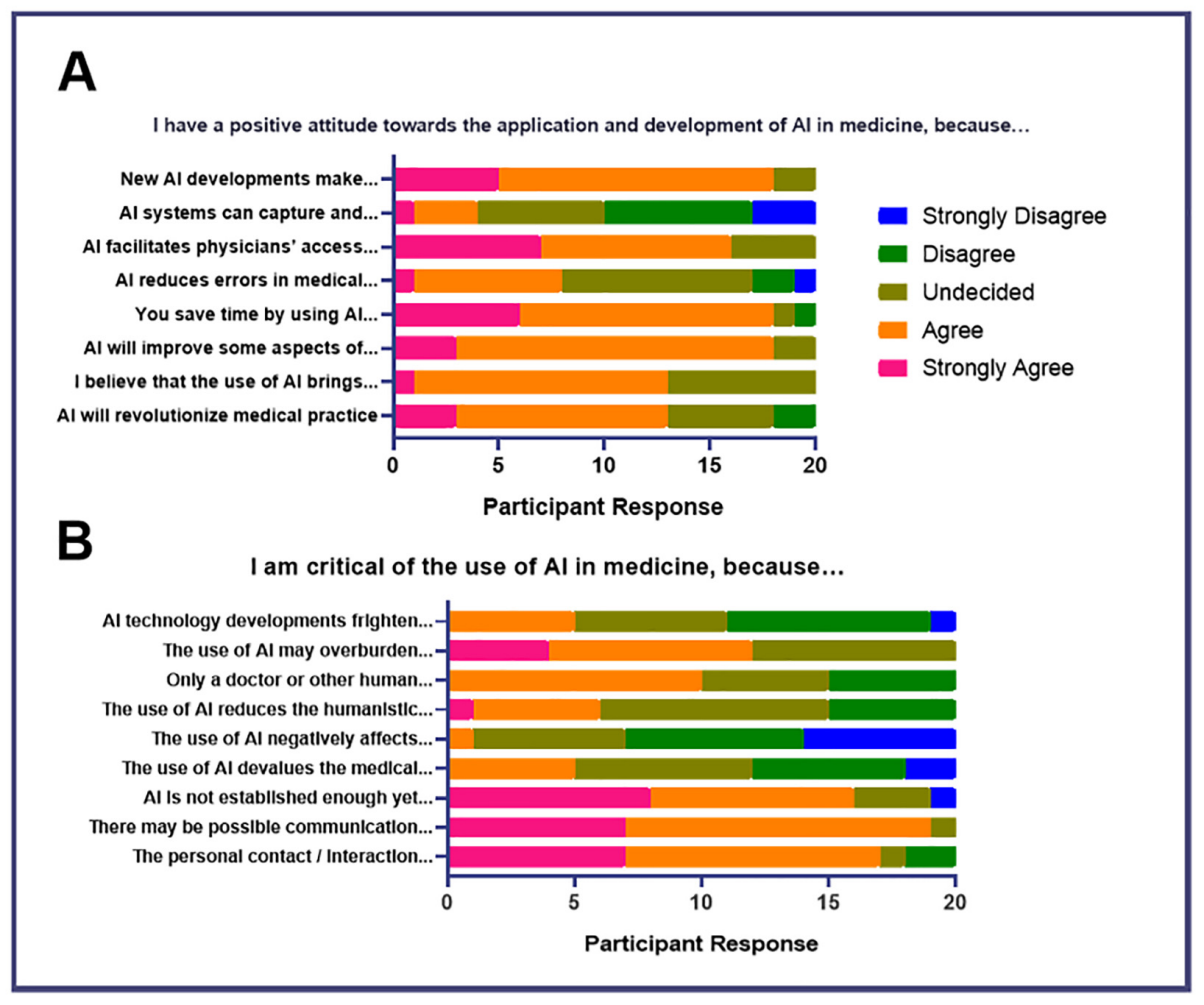
Attitudes of medical students toward AI in medicine (Impact of AI on Doctor’s Role).

The majority of respondents express a positive attitude towards the application and development of AI in medicine because it may make the field more exciting, facilitate access to information, save time, improve some aspects of healthcare, and revolutionize medical practice ( Figure 2A). These responses are supported by the group's sentiments on the advantages of using artificial intelligence (Extended Data 4) where most agreed that it can speed up healthcare processes and deliver real-time, clinically-relevant, high-quality data. The majority also said that its use would be beneficial since AI has no space-time constraints, emotional exhaustion, or physical limitation. However, 45% ( *n*=9) of respondents are undecided regarding its capacity to reduce errors in medical practice while 15% ( *n*=3) expressed disagreement with this idea ( Figure 2A). This is inconsistent with later responses where most respondents still believed that AI may be advantageous as it can help reduce medical errors, reflecting possible uncertainties due to insufficient education of the applications of AI in medical practice (Extended Data 4).

Moreover, the participants generally disagree or are undecided that AI can make a diagnosis with greater accuracy and speed ( Figure 2A). This is supported by the responses of the group regarding their reservations about the use of AI (Extended Data 4). The majority stated that only a human healthcare professional can make the right decision about patient management. Aside from this, the group is critical of the use of AI because its role in Medicine has not been established which may create problems in communication and the physician-patient interaction. Furthermore, 50% ( *n*=10) of respondents believe that it may remove the humanistic aspect of the practice of Medicine. Generally, respondents do not believe that AI can do a better job than human beings and disagree that its diagnostic ability is superior to the clinical experience of doctors ( Figure 2B). All respondents believe that AI cannot be used in unpredicted situations because its applications may be limited in controversial topics. Most respondents stated that it may not apply to all patients ( *n*=16, 80%), it had less ability to consider the emotional well-being of the patient ( *n*=16, 80%), and it may have inadequate information regarding the patient’s situation ( *n*=11, 55%). Meanwhile, half of the participants responded that it may not be applicable in unprecedented situations because it may have been developed by a specialist who may not have the same level of clinical experience as a human physician.

### Impact on the doctor-patient relationship

**Figure 3.**
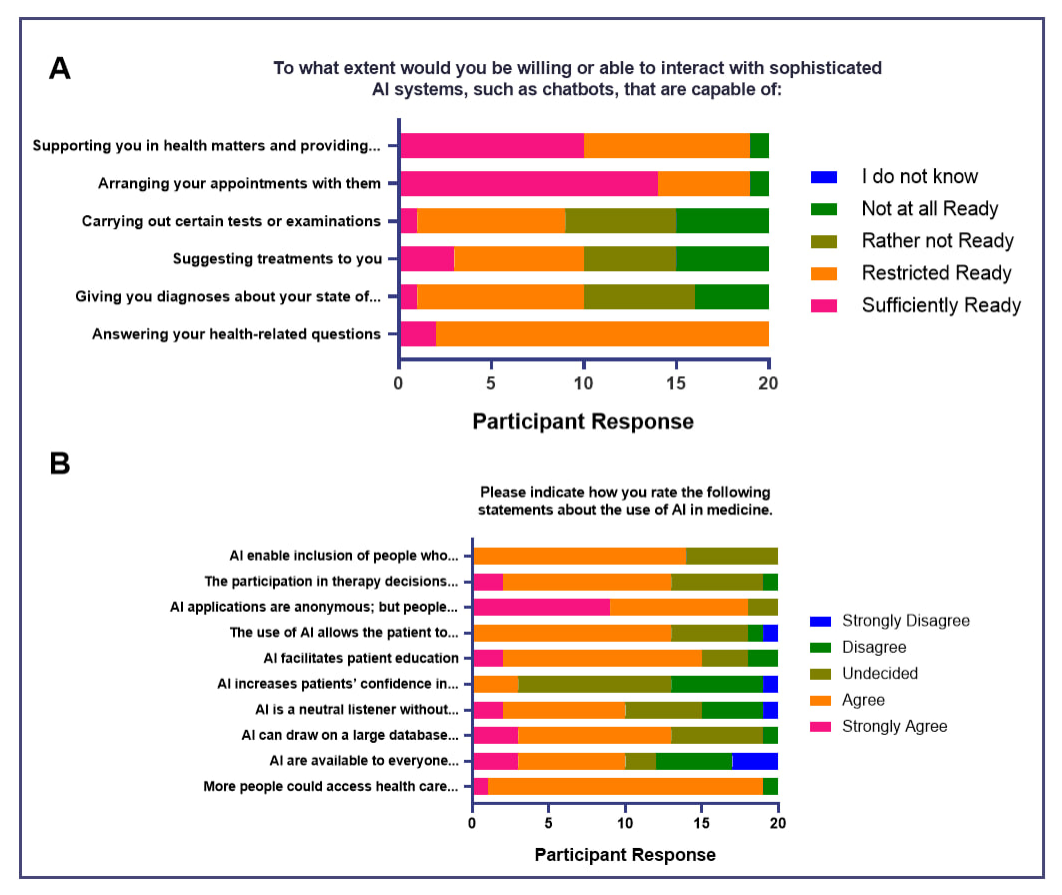
Attitudes of medical students toward AI in medicine (Impact of AI on Doctor-Patient Relationship).

The survey results indicate varying degrees of readiness among the respondents to interact with sophisticated AI systems for different healthcare-related tasks ( Figure 3A). With regards to AI systems that can answer health-related questions, a majority ( *n*=18, 90%) are restrictedly ready, implying openness to this functionality. When it comes to AI systems that are capable of arranging appointments and providing additional health information, a significant portion of respondents are ready, pointing to a favorable reception of the functions. Opinions are divided regarding AI systems that are capable of giving diagnoses and suggesting treatments, implying tentativeness to these operations.

Majority of the respondents ( *n*=19, 95%) believe that more people could access health care more conveniently and quickly through the use of AI ( Figure 3B). Opinions are more divided regarding the availability of AI (e.g., chatbots, virtual assistants) regardless of time and location, with 50% agreeing of its accessibility, and 40% ( *n*=8) of respondents believing otherwise. With regards to the sentiment of AI being able to have a wider knowledge base than physicians, and that it may facilitate patient education and allow the patient to increase his control over his own health, more than half agree. A majority ( *n*=14, 70%) also believe that AI enables the inclusion of people who may find it difficult to confide in a doctor, relating to half of the respondents agreeing that AI is a neutral listener and is not personally judgmental. However, half of the group is still undecided regarding whether AI increases patients’ confidence in medicine. A large part of the group ( *n*=18, 90%) agree that people still need a human counterpart when it comes to their health care.

The results suggest that as the respondents acknowledge the benefits of AI in acquiring knowledge regarding health and medicine, the group highlights that human interaction is still essential in medical practice. Moreover, while the respondents are generally open to AI accomplishing supportive and administrative tasks in medical practice, the group is more wary of AI taking on diagnostic and treatment-related roles.

### Ethical concerns for AI use in medicine

**Figure 4.**
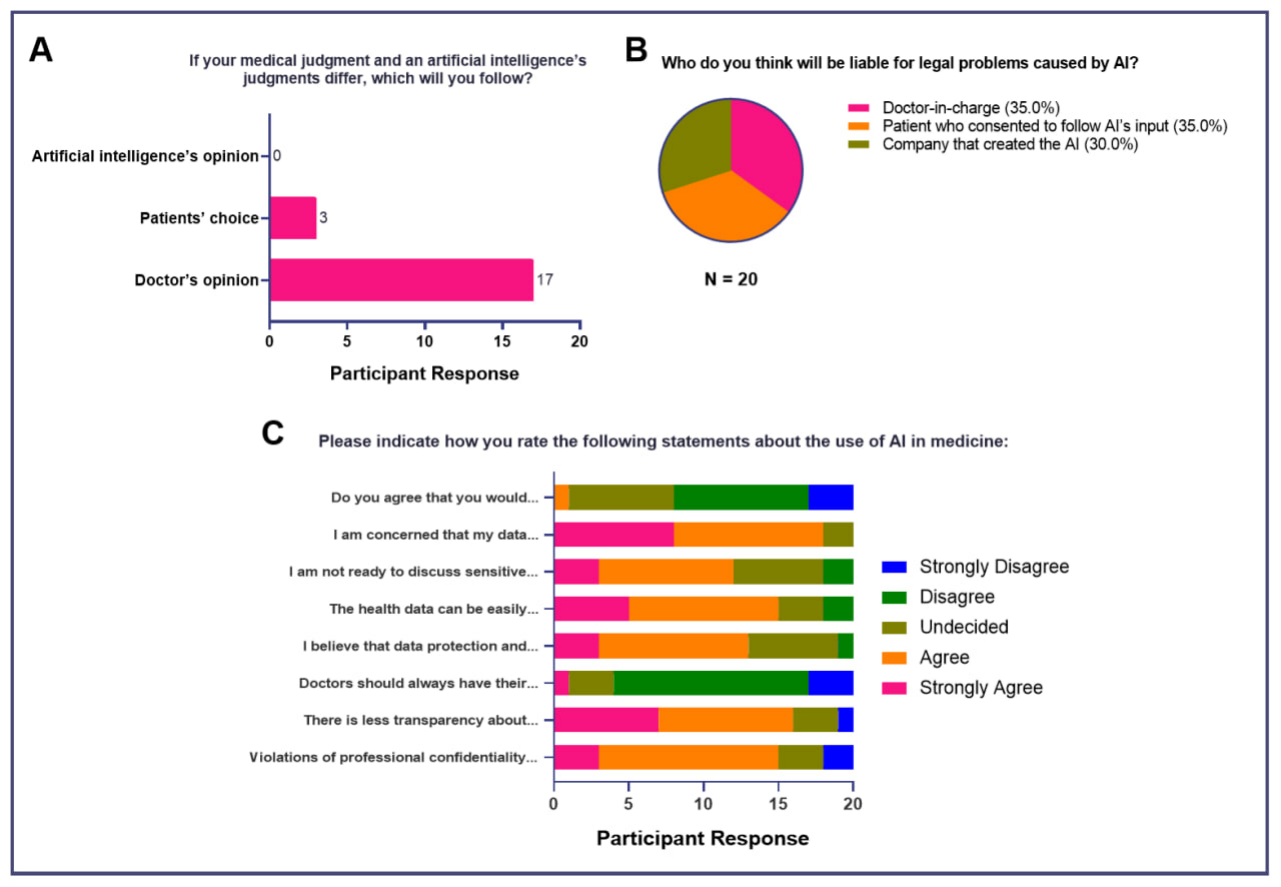
Attitudes of medical students toward AI in medicine (Ethical Considerations for Use of AI in Medicine).

In terms of medical judgment, most agreed that the doctor’s opinion took precedence ( *n*=17, 85%), followed by the patient's choice when it came to medically-relevant decision-making ( *n*=3, 15%). Interestingly, none of the study participants voted AI’s opinion as the primary basis for decision-making ( Figure 4A). When asked about liability for medico-legal problems caused by AI, 30% agreed that the doctor-in-charge ( *n*=7) and the patient who consented to follow the AI’s output ( *n*=7) held equal responsibility. However, the company that created and developed the AI can also be held responsible, as well ( *n*=6, 30%) ( Figure 4B). Finally, when asked about various ethical dilemmas, the majority either strongly agreed or agreed that concerns related to data privacy ( *n*=13, 65%), data manipulation ( *n*=15, 75%), discussion of sensitive data with AI ( *n*=12, 60%), violations of professional confidentiality ( *n*=15, 75%), and transparency may arise with use of AI in clinical practice ( *n*=16, 80%). Moreover, the majority disagreed that doctors should have their diagnoses checked by AI ( *n*=13, 65%) and 45% ( *n*=9) stated that future physicians should likewise not be reliant on AI when making medical decisions.

### Focus group discussion

Based on the results of the focus group discussions conducted, three recurring themes underscored the discussions across both groups - (1) on comparing AI to human intelligence, (2) on the use of AI as a versatile tool to enhance and augment learning, and (3) on the insufficiency of training being provided for medical students and educators alike on the use of AI ( Table 1).

**Table 1.** Overview of categories, subcategories, and corresponding sample excerpts based on latent inductive thematic analysis ( Braun & Clarke, 2023; Byrne, 2022).

Category	Subcategory	Example
Comparisons of AI vs. Human Intelligence	Human Intelligence is more robust and capable of generating novel ideas	AI utilizes pre-programmed data to characterize phenomena, employing pattern recognition from datasets. Its outputs are determined by its inputs; thus, it is limited by the information it receives.
Comparisons of AI vs. Human Intelligence	AI is less prone to semantic and technical errors	AI is able to remain impartial and free from emotional biases inherent in humans. By operating on datasets, AI enhances the reliability and validation of information that is inputted, promoting accuracy in its analyses.
Comparisons of AI vs. Human Intelligence	AI cannot be humanized and cannot replace physicians	AI does not have the ability to interpret non-verbal cues (such as gestures, tone of voice, facial expressions) as its processing is limited to the information directly provided to it.
AI as a versatile tool for augmenting learning and teaching	AI can be designed to cater towards a specific learning outcome or objective	Another opportunity that we can explore is in summarizing. I’ve tried to ask an AI to summarize a book or research article and I noticed that it could not. Some people use it to automate making learning assessments (e.g., flashcards). However, it can give wrong information that’s why when you use it, you must have some level of literacy.
AI as a versatile tool for augmenting learning and teaching	AI can enable more creative and stimulating teaching paradigms and learning environments	Thinking about AI more broadly, not just the language AIs. AIs could be trained. Not just chatbots, but machines that simulate characters are also AI.
Insufficiency of training in AI in the medical curriculum	Setting realistic expectations for what can be achieved with AI	It needs to be more applicable to use because we’re under community-oriented medical orientation, so we specifically cater to the Philippine community.
Insufficiency of training in AI in the medical curriculum	Addressing issues of plagiarism and unreliable sources of information	Always remember that the information is not always right. The danger is that you might be over reliant with AI. You should countercheck with more credible sources.
Insufficiency of training in AI in the medical curriculum	Potential data privacy violations with use of AI and storage of personal data	Data privacy – how data is stored. When we input something, we don’t know if there are 3rd parties involved. When you study medicine, patient data can be put into AI but we do not know where the data will be stored.

Focus group discussions with the student participants of the study revealed three major themes concerning the role of AI in medical education and the practice of medicine as a whole ( Table 1). Firstly, a distinction between the capabilities and role of human intelligence and AI must be delineated clearly. Human intelligence is shown to be more robust and capable of innovation, more than AI but AI has inherent limitations preventing it from becoming fully humanized and capable enough to replace human physicians in the healthcare field. However, AI does present some advantages in being less prone to semantic and technical errors. This leads to the second major point of AI being a versatile tool for augmenting learning and teaching in the medical education setting. More specifically, AI can be tailored towards achieving specific learning outcomes or objectives and utilized to form stimulating and engaging teaching strategies and learning settings for medical students. However, the level of training on AI provided in the current medical curriculum is still inadequate to enable effective and responsible use of these tools. This was further evidenced by the answers made by the study participants in the earlier survey conducted, which showed the majority still felt that their level of knowledge of AI was insufficient, even after having taken a course on medical informatics ( Figure 1). Key areas which need to be addressed include the ability to set realistic expectations of AI as a tool for learning and addressing issues related to academic plagiarism, unsatisfactory information sources, data breach, and exploitation of personal data among others.

## Discussion

The use of AI in various training institutions is often a point of discussion in contemporary educational environments, due to how it shapes the mindset of current physicians-in-training and its effect on their eventual practice as healthcare providers. In this study, we aimed to provide a better understanding regarding the perceptions of medical students on the use of AI and the inclusion of AI in the medical education system. Moreover, we intended to explore the different aspects that must be integrated in current curricular didactics.

The survey and focus group discussions revealed that most participating students have a baseline awareness and understanding of AI and how it works in the context of medical education and practice. Moreover, students showed interest in learning more about the applications of AI in medicine. However, its role in acquiring knowledge and skills necessary to become competent healthcare professionals remains poorly understood. As seen in the survey and highlighted in the focus group discussion, AI may be a versatile tool for augmenting learning and teaching but the training available to Filipino medical students through the current medical curriculum is insufficient due to the accessibility and applicability of AI tools in the country as well as the ethical issues surrounding the use of AI. In particular, students elaborated that the ethical issues of concern include academic plagiarism, unsatisfactory information sources, breaches in data privacy, and exploitation of personal data. These suggest that learning institutions may need to become more proactive in introducing the responsible use of AI in medicine.

These findings are consistent with what was observed in other similar studies on similar topics which recommended that appropriate intervention in the form of dedicated modules or short lectures on the role of AI in clinical practice be done ( Basu *et al*., 2020). Furthermore, a number of challenges have been identified in previous studies related to the utility of AI in medical education including (1) ethical and legal concerns, (2) limitations in the scalability of application, (3) proper evaluation of the effectiveness of teaching methods for learners, and (4) issues related to technical difficulties and technological limitations ( Zarei *et al*., 2024). In the same study, the authors emphasized the need for periodic evaluations of the effectiveness of academic curricula based on identified needs and challenges, meriting further investigation.

The present study illustrates the need to address the absence of dedicated courses on the responsible use of AI in the current Philippine medical curriculum. This gap is worsened by several challenges present in Philippine healthcare and educational systems. Underdeveloped technological infrastructure, especially in underserved areas, restricts exposure to digital technologies such as AI.

Training gaps in ethics further exacerbate these challenges. Many Philippine medical schools do not have modules on the ethical use of AI, reflecting broader gaps in ethics education. These training gaps must be addressed, as ethical challenges such as academic integrity and data privacy were frequently cited by participants in focus group discussions and other literature ( Alowais *et al*., 2023).

Systemic barriers such as resource constraints, regulatory frameworks, and curricular rigidities further hinder the effective integration of AI into medical education ( Guignona *et al*., 2021). The integration of new technologies often requires navigation into complex bureaucratic processes and overcoming limited infrastructure, further hindering educators and institutions from integrating AI into their teaching and leaving students with limited avenues to explore the applications and responsible use of AI in medicine. These contextual challenges highlight the need for initiatives such as curriculum reforms, and investment in infrastructure tailored to the Philippine setting. Addressing these systemic issues can enable medical institutions to empower their students to engage with AI responsibly and effectively.

Considering the emerging impact of AI in clinical practice, particularly in diagnosing diseases ( Becker *et al*., 2022; Graham *et al*., 2019; Han *et al*., 2020; Kim *et al*., 2020; Wang & Avillach, 2021), researching treatment ( Sheu *et al*., 2023), developing plans for patient management ( Nelson *et al*., 2019), implementing public health programs, and educating patients, the role of AI on career choices among medical students, on the roles of physicians, and on the physician-patient relationship were explored ( Alowais *et al*., 2023).

 Our study showed that the emerging role of AI did not impact the participating medical students’ decisions regarding their medical career, although they were aware of the possible effects of AI on certain fields and specializations. A systematic review by Sun *et al*., in particular, highlights how most fields related to radiology, diagnostics, surgery, and cardiology were the primary areas of focus in terms of applying AI to medical training. These findings were consistent with our study, although surprisingly, disciplines such as Family and Community Medicine and Psychiatry were also indicated as specializations affected by AI, possibly due to the emerging role of virtual assistants and mental health chatbots ( Moldt *et al*., 2023; Sun *et al*., 2023). However, in our study, participating students expressed that the applications of AI in fields such as community medicine may be limited due to the unequal access to technology in the Philippines.

In terms of concrete action points, our study suggested exploring different ways to utilize AI in various didactics to achieve learning outcomes. For example, integrating the use of AI to process learning materials in the form of adaptive learning systems and intelligent tutoring systems can help augment the overall classroom experience for adult learners. This can come in the form of simulation assessments, virtual patients, and management of learning resources ( Narayanan *et al*., 2023; Weidener & Fischer, 2024). As highlighted in the present study, medical students have already begun exploring the application of the tool in their studies in various ways (i.e., reading medical literature, developing differential diagnoses, interpreting laboratory tests). Therefore, it is imperative for learning institutions and educators to be sufficiently equipped with ways to consider and effectively implement AI into their teaching strategies in response to these tools becoming more available to students ( Alowais *et al*., 2023). Rather than barring the use of AI, many studies have come to the conclusion that responsible use of AI applications while ensuring the protection of professional rights and values must become the primary approach to its integration in the medical educational system ( Alowais *et al*., 2023; Civaner *et al*., 2022)

Finally, more focus must be placed on elaborating on the role of AI in healthcare provision and in the patient-doctor relationship, specifically on how the tool can be used to enhance patient care while minimizing concerns related to data privacy, bias, and the importance of human expertise. This is an equally important point to consider since patients are able to access these tools just as much as medical students and professionals are able to, as well. Therefore, to address ethical dilemmas which may arise from irresponsible or unregulated use of AI on the side of the patient, healthcare practitioners must be sufficiently equipped with the means necessary to engage in proper discussion with patients who utilize these resources available to them. This would, in turn, foster and invoke the humanistic aspect of patient care that was raised as a point of concern in the current study and is still an ongoing point of discussion among experts in the field ( Lee *et al*., 2021).

## Limitations

The scope of this study was limited to the exploration of the perceptions and attitudes of medical students to AI in medical education. Perspectives of medical faculty, allied medical professionals and students, and students of other disciplines not related to medicine were not explored in the present study. The learning stage of the study participants ensured that all members had previously heard of the tool and were already exposed to the field of medicine, but opinions made may not be fully evidenced and primarily serve as preliminary basis for future investigations in the field. Additionally, study participants were from the University of the Philippines College of Medicine. This may limit the depth and breadth of the findings gathered since this may not sufficiently cover ideas from other medical learning institutions that adopted other medical educational models. The study is limited to descriptive statistics. Subgroup analyses such as possible factors influencing the students’ attitudes were not done as the cohort were all from the same institution and country. Moreover, although the role of AI in medical practice was investigated and discussed, this was not elaborated on in full detail since this requires further exposition on the impact of AI on the doctor, the patient, and the doctor-patient relationship, as a whole. Findings of the study shall mainly be used in suggesting learning competencies and objectives necessary for a medical student to gain a better understanding and appreciation for the role of AI in medicine.

## Conclusion

In conclusion, our findings show that medical students have developed a baseline understanding and awareness of AI but require more opportunities to formally train and learn about responsible use of these tools, in both the medical education and, eventually, clinical practice landscape. Such courses must be designed to sufficiently equip students with the fundamental knowledge needed to overcome ethical concerns related to AI use and utilize these tools in practical applications to improve patient care and healthcare provision, altogether. However, given the limited sample size of the present work, it is important to recognize that further investigations must attempt to explore the perceptions or attitudes of larger cohorts of students in order to better evaluate the overall state of educational needs of medical students in terms of AI-related competencies.

## Ethics and consent statement

The study received ethical clearance from the University of the Philippines Manila Research Ethics Board (UPMREB 2024-0345-EX) and adhered to the WMA Declaration of Helsinki – Ethical Principles for Medical Research Involving Human Participants ( World Medical Association, 2024). Participation was voluntary, and students provided their written informed consent and received no compensation. Each study participant was adequately informed of the aims, methods, sources of funding, any possible conflicts of interest, institutional affiliations of the researcher, the anticipated benefits and potential risks of the study and the discomfort it may entail, post-study provisions and any other relevant aspects of the study. Participants were informed of the right to refuse to participate in the study or to withdraw consent to participate at any time without reprisal. All responses and data were anonymized and confidentiality was assured.

## Data Availability

FigShare: Exploring Filipino Medical Students’ Attitudes and Perceptions of Artificial Intelligence in Medical Education: A Qualitative Mixed-Methods Study: https://doi.org/10.6084/m9.figshare.26506837.v2 ( Falcon *et al*., 2024) This project contains the following underlying data: Figure 1. Attitudes of medical students toward AI in medical education (Educational Needs Assessment). Figure 2. Attitudes of medical students toward AI in medicine (Impact of AI on Doctor’s Role). Figure 3. Attitudes of medical students toward AI in medicine (Impact of AI on Doctor-Patient Relationship). Figure 4. Attitudes of medical students toward AI in medicine (Ethical Considerations for Use of AI in Medicine). Data are available under the terms of the Creative Commons Attribution 4.0 International license (CC-BY 4.0). FigShare: Exploring Filipino Medical Students’ Attitudes and Perceptions of Artificial Intelligence in Medical Education: A Qualitative Mixed-Methods Study: https://doi.org/10.6084/m9.figshare.26506837.v2 ( Falcon *et al*., 2024) This project contains the following underlying data: Extended Data 1. Raw anonymized survey results. Extended Data 2. Baseline knowledge of AI in medicine and sociodemographic characteristics of participants. Extended Data 3. Attitudes of medical students towards AI in medicine. Extended Data 4. Attitudes of medical students toward AI in medicine (Impact of AI on Doctor’s Role). Extended Data 5. Attitudes of medical students toward AI in medicine (Impact on Career). Extended Data 6. Transcript of focus group discussion group 1 Extended Data 7. Transcript of focus group discussion group 2 Data are available under the terms of the Creative Commons Attribution 4.0 International license (CC-BY 4.0).

## Software availability statement

Descriptive statistical analysis of the data collected through the questionnaires was performed using GraphPad Prism version 8.0.1 for Windows, GraphPad Software ( www.graphpad.com). Alternatively, figures may also be generated with RStudio version 4.2 ( https://www.R-project.org/).
