# Supplementary material for: Exploring Filipino Medical Students’ Attitudes and Perceptions of Artificial Intelligence in Medical Education: A Mixed-Methods Study
Source: MedEdPublish (2016). 2025 Apr 1;14:282. Originally published 2024 Nov 20. [Version 2] doi: 10.12688/mep.20590.2 (PMC12082071; doi:10.12688/mep.20590.2)
Supplement: Peer review history

# Reviewer report: Reviewer response for version 2

**DOI:** 10.21956/mep.22477.r41643

**Published:** 2025-05-13

**Authors:** Nilesh Kumar Mitra

**Affiliations:** 1 IMU University, Kuala Lumpur, Malaysia

The authors indicated in the introduction section that the study aimed to provide an overview of current attitudes and perceptions of Filipino medical students enrolled in a course. The authors also outlined the study's specific objectives:

 i. To understand how AI technologies work and how these technologies affect the understanding of medical students in healthcare provision, and the future of medicine.

 ii. To determine the attitudes of medical students about the use of AI in the medical curriculum.                  

 Methods

 The authors used 24 questions with 5 sub-sections (quantitative study) and 10 questions for focus group discussions (qualitative study). Only 20 pre-clinical students from an elective course were participants in the study. 

 a) The title of the study : "Exploring Filipino Medical Students' Attitudes and Perceptions of AI in Medical Education: A Mixed-method study"

 b) In relation to the title and stated objectives of the study, the design of the study should have included proportionate numbers of medical students by application of a sampling frame (probability sampling or non-probability sampling) as well as by the use of sample size calculation with stated aim of power of statistical analysis. 

 c) As stated by the other reviewer, the sample size of twenty is not able to achieve the objective of the quantitative study component stated in the introduction. Martin (2018) suggested a minimum thirty-person sample size for each quantitative research variable [1].

 d) The authors had the limitation of not being able to apply analytical statistics and state in the methodology due to a limited number of samples used in the study.

 e) Although some validation exercises have been done, the type of thematic analysis (with citation) employed in the qualitative study is not available.

 Results

 We have noted in the objectives of the study that the authors aimed to understand how AI technologies work. The results section is not clear about the technologies being used by these pre-clinical students. From previous published studies, it has been observed that the pre-clinical students often use AI tools for translation, summarization, assessment tool generation, clinical scenario generation and literature review for intended research. Use of Generative AI for the preparation of PBL has also been the topic of several recent research studies. 

 I also want to comment on the survey results indicating varying degrees of readiness among the respondents to interact with sophisticated AI systems for different healthcare-related tasks.

 As stated in the methodology: students were in the pre-clinical stage of their medical education, primarily focusing on foundational sciences with limited clinical exposure.

 The gap in the study design is well visible. Can the prospective reader believe about the survey result of varying degrees of readiness to interact with sophisticated AI systems for healthcare-related tasks, when the sample under the study has little experience in participating in healthcare-related task.

 The same applies to the results mentioned under impact on carrier with pre-clinical students having little knowledge of medical carrier

 Discussion

 Discussion should include the analysis about how the inclusion of pre-clinical students as sample has affected the results and objectives of the study. The authors should try to compensate by bringing in perspectives from the similar studies conducted with clinical students. 

 Conclusion

 The conclusion is written too simply without including the significant findings. It should also include the limitations discussed above.

Have any limitations of the research been acknowledged?

Yes

Is the work clearly and accurately presented and does it cite the current literature?

Yes

If applicable, is the statistical analysis and its interpretation appropriate?

No

Are all the source data underlying the results available to ensure full reproducibility?

Yes

Is the study design appropriate and is the work technically sound?

No

Are the conclusions drawn adequately supported by the results?

No

Are sufficient details of methods and analysis provided to allow replication by others?

No

Reviewer Expertise:

Technology enhanced learning

I confirm that I have read this submission and believe that I have an appropriate level of expertise to confirm that it is of an acceptable scientific standard, however I have significant reservations, as outlined above.

# Reviewer report: Reviewer response for version 2

**DOI:** 10.21956/mep.22477.r41322

**Published:** 2025-05-13

**Authors:** Peih-Ying Lu

**Affiliations:** 1 Kaohsiung Medical University Hospital, Kaohsiung, Taiwan

Thank you for the authors' detailed responses. I appreciate the efforts. However,  some issues require further clarification:

 1. Contextualizing Equity and Integration Barriers in the Philippines.

 Comment: Needs deeper contextualization within local systems.

 *Partially addressed. It’s unclear whether these contextual elements were explicitly connected to their findings or remained general. Please tie contextual barriers directly to specific survey or FGD findings for greater analytical depth.

 2. Link to Philippine Medical Education Barriers Comment: More robust justification using local educational challenges.

 Authors’ Response: Added references to global literature and touched on local factors.

 *Insufficient unless they explicitly engage with Philippine-specific barriers, such as infrastructure gaps, faculty tech training deficits, etc. Please provide some clearer grounding in systemic local issues.

 3. Lack of Inferential Statistics and Comparative Insight

 Comment: Solely descriptive statistics, no comparison across groups.

 Authors’ Response: Justified as exploratory; added global comparisons in discussion.

 *Please acknowledge this as a limitation and consider stratified or comparative analysis in future studies.

 4.  Quant–Qual Integration

 Comment: Insufficient integration between datasets; triangulation unclear.

 Authors’ Response: Cited Guetterman’s approach and described meta-inference.

 *The authors explain *what they did* but not *how it actually looked*. Please shows how a theme is supported by both survey data and FGD quotes.

 5.  Ethical Concerns Underexplored in FGDs

 Comment: Ethical issues need to be probed more in qualitative data.

 Authors’ Response: Noted for future work; not addressed in this round.

 *Not adequately addressed. While it’s understandable for a pilot, the authors should at least note this as a current limitation and describe how it will be rectified in follow-up studies.

 6.  Qualitative Analysis Method Not Specified

 Comment: Unclear if thematic analysis was used.

 Authors’ Response: Used inductive content analysis; cited Braun & Clarke.

 *Inconsistent. If the authors used Braun & Clarke, that’s *thematic analysis*, not *content analysis*. This contradiction should be clarified — either they used one or the other. Please align terminology and explain why that approach was selected.

Have any limitations of the research been acknowledged?

Partly

Is the work clearly and accurately presented and does it cite the current literature?

Yes

If applicable, is the statistical analysis and its interpretation appropriate?

Partly

Are all the source data underlying the results available to ensure full reproducibility?

Partly

Is the study design appropriate and is the work technically sound?

Partly

Are the conclusions drawn adequately supported by the results?

Partly

Are sufficient details of methods and analysis provided to allow replication by others?

Partly

Reviewer Expertise:

medical humanities, professionalism, globalizaion and cultural studies, curriculum design

I confirm that I have read this submission and believe that I have an appropriate level of expertise to confirm that it is of an acceptable scientific standard, however I have significant reservations, as outlined above.

# Reviewer report: Reviewer response for version 2

**DOI:** 10.21956/mep.22477.r41321

**Published:** 2025-04-07

**Authors:** Ken Masters

**Affiliations:** 1 Sultan Qaboos University, Muscat, Muscat Governorate, Oman

The authors have adequately addressed most of my concerns.

Have any limitations of the research been acknowledged?

Partly

Is the work clearly and accurately presented and does it cite the current literature?

Partly

If applicable, is the statistical analysis and its interpretation appropriate?

Partly

Are all the source data underlying the results available to ensure full reproducibility?

Yes

Is the study design appropriate and is the work technically sound?

Yes

Are the conclusions drawn adequately supported by the results?

Partly

Are sufficient details of methods and analysis provided to allow replication by others?

Yes

Reviewer Expertise:

Medical Informtaics, AI.

I confirm that I have read this submission and believe that I have an appropriate level of expertise to confirm that it is of an acceptable scientific standard.

# Reviewer report: Reviewer response for version 1

**DOI:** 10.21956/mep.22039.r40857

**Published:** 2025-01-27

**Authors:** Ken Masters

**Affiliations:** 1 Sultan Qaboos University, Muscat, Muscat Governorate, Oman

This is a very small and localised study of (non-AI expert) medical students’ attitudes towards AI.  The size limitation is, however, overcome to some extent by the use of focus groups, which give greater insight into the issues.  Although the generalisability is uncertain outside of the local context, there is still value for all readers.  Because of this limitation, however, the paper does need to make it clear that conclusions and inferences are limited.  This is addressed in more detail in my comments below.

**Some points that should be addressed by the authors:**

1. In the Abstract, the Results section needs to show more detail.  Please give some of the most important statistics.
2. Given that the Declaration of Helsinki has recently been updated (2024) ( https://www.wma.net/policies-post/wma-declaration-of-helsinki/), I would recommend that the authors update their citation and reference.
3. Some of the paragraphs are very long and need to be broken up into smaller, manageable pieces.
4. The statistics contained in the paragraph beginning “When asked to which degree should certain AI-related topics…” should rather be in a table for ease of reading.
5. The participants are not expert in AI, and their only training in his field is a single elective course.  As a result, many of the opinions offered in the Focus Groups (e.g. comparison of AI and human intelligence) are opinions of people who are not experts in either AI or the study of human intelligence.  So, while those opinions are useful for the institution, they are not necessarily insights into the topics themselves; rather they identify the students’ pre-conceived ideas, but that is as far as their value goes.  This should be noted when presenting the Focus Group results and also in the Limitations.
6. Related to this, the Discussion needs to be more cautious in its statements about the study’s implications.  Although the fact that is a small and localised study is mentioned in the Limitations, this needs to be referred to in the Discussion so that the authors cannot be accused of making unfounded generalisations.
7. This needs to be carried over the Conclusion also.

Have any limitations of the research been acknowledged?

Partly

Is the work clearly and accurately presented and does it cite the current literature?

Partly

If applicable, is the statistical analysis and its interpretation appropriate?

Partly

Are all the source data underlying the results available to ensure full reproducibility?

Yes

Is the study design appropriate and is the work technically sound?

Yes

Are the conclusions drawn adequately supported by the results?

Partly

Are sufficient details of methods and analysis provided to allow replication by others?

Yes

Reviewer Expertise:

Medical Informtaics, AI.

I confirm that I have read this submission and believe that I have an appropriate level of expertise to confirm that it is of an acceptable scientific standard, however I have significant reservations, as outlined above.

# Response

**Published:** 2025-03-22

**Authors:** Robbi Miguel Falcon

**Affiliations:** College of Medicine, University of the Philippines Manila, Manila, Metro Manila, Philippines

We would like to thank the reviewer for taking their time and effort to review the manuscript, once again. We greatly appreciate your valuable suggestions and comments to improve the quality of the manuscript. We explain here how we edited and addressed the concerns and corrections you raised in your review, as well as the additional edits we thought were necessary to improve the paper. The revisions and answers are highlighted in red font in the manuscript. This is a very small and localized study of (non-AI expert) medical students’ attitudes towards AI.  The size limitation is, however, overcome to some extent by the use of focus groups, which give greater insight into the issues.  Although the generalizability is uncertain outside of the local context, there is still value for all readers.  Because of this limitation, however, the paper does need to make it clear that conclusions and inferences are limited.  This is addressed in more detail in my comments below. Discussion and Conclusion The statements made in the study were revised and supported by previous studies, with similar findings to improve validity of claims.   A statement on the limited applicability of the findings of the study due to the limited sample size was included.    

**Comment: **In the Abstract, the Results section needs to show more detail.  Please give some of the most important statistics. Given that the Declaration of Helsinki has recently been updated (2024) (https://www.wma.net/policies-post/wma-declaration-of-helsinki/), I would recommend that the authors update their citation and reference.

**Response:** 2.2 Ethical Considerations The section on the ethical considerations of the study was revised.

**Comment: **Some of the paragraphs are very long and need to be broken up into smaller, manageable pieces.

**Response:** Several paragraphs were made shorter and broken down into smaller, manageable blocks of text.

**Comment: **The statistics contained in the paragraph beginning “When asked to which degree should certain AI-related topics…” should rather be in a table for ease of reading.

**Response:** Figure 1 The statistics were already included in Figure 1. 

**Comment:** The participants are not expert in AI, and their only training in his field is a single elective course.  As a result, many of the opinions offered in the Focus Groups (e.g. comparison of AI and human intelligence) are opinions of people who are not experts in either AI or the study of human intelligence.  So, while those opinions are useful for the institution, they are not necessarily insights into the topics themselves; rather they identify the students’ pre-conceived ideas, but that is as far as their value goes.  This should be noted when presenting the Focus Group results and also in the Limitations.

**Response: **Discussion, Limitations: This comment is well-noted and received, and the limitations of the study participants’ opinions were highlighted in the text.

**Comment: **Related to this, the Discussion needs to be more cautious in its statements about the study’s implications.  Although the fact that is a small and localized study is mentioned in the Limitations, this needs to be referred to in the Discussion so that the authors cannot be accused of making unfounded generalizations.

**Response:** Discussion: The statements made in the study were revised and supported by previous studies, with similar findings to improve validity of claims.

**Comment:** This needs to be carried over the Conclusion also.

**Response:** Conclusion: A statement on the limited applicability of the findings of the study due to the limited sample size was included. Thank you very much!

# Reviewer report: Reviewer response for version 1

**DOI:** 10.21956/mep.22039.r40217

**Published:** 2024-12-19

**Authors:** Peih-Ying Lu

**Affiliations:** 1 Kaohsiung Medical University Hospital, Kaohsiung, Taiwan

This study aims to explore Philippino attitudes and perceptions of AI.  The research questions align with global trends in integrating AI into medical education, a pressing issue as technology rapidly transforms healthcare systems. But at the same time, it’s also a complicated issue to address, particularly in some developing countries that are facing other issues, such as equitable AI use. The study highlights a gap in formal training on AI within medical curricula in the Philippines. The research questions in the article are justified to a certain extent, but there are areas where they could be more rigorously framed or contextualized.

1. The research questions are quite broad, attempting to cover attitudes toward AI in education, its application in clinical practice, and ethical concerns. This breadth risks diluting the focus, especially given the small sample size and limited institutional scope.
2. Questions about AI's impact on specific aspects of medical practice (e.g., diagnostics, doctor-patient interactions) could be better justified with prior literature or hypotheses.
3. The paper briefly mentions AI equity and challenges in AI integration in developing countries but needs to deeply contextualize these challenges within the Philippine healthcare and education systems.
4. A stronger linkage to specific barriers in the Philippine medical education context (e.g., resource limitations and faculty readiness) would provide a more robust justification. While the study is pioneering in the Philippine context, the research questions are similar to those explored in other global studies. Cultural attitudes toward technology or systemic challenges in medical training can also be provided.

**Methodology:**

1. The participants were 20 students, and the sample of 20 students was inadequate for quantitative analysis, especially for generalizing findings—the participants' demographic needs to be provided, such as the learning stage of this cohort.
2. The study did not provide more details about the validation of the questionnaire.
3. The study relies solely on descriptive statistics without exploring relationships or differences using inferential statistics. This limits deeper insights into the data, such as identifying factors influencing attitudes. There is no comparison with other cohorts, such as students from different institutions or countries, which would help contextualize the findings and highlight unique aspects of the Filipino medical education system.
4. The focus group questions are not directly mapped to specific quantitative survey questions or subsections. This makes it harder to draw precise links between the datasets. How the qualitative data was triangulated with the quantitative one needs to be elaborated. In other words, how the qualitative data complemented the quantitative data requires further explanation.
5. While ethical concerns are a prominent theme in the survey, they need to be sufficiently emphasized in the focus group questions. For example, data privacy and liability concerns mentioned in the quantitative section could have been probed further in the qualitative discussions.
6. The analysis of qualitative data (interviews) needs to be more clarified. For instance, did the authors use thematic analysis?

**Discussion:**

 The discussion section effectively summarizes the study’s key findings and aligns with previous literature. However, while the study uses a mixed-methods approach, the discussion does not fully integrate insights from the quantitative and qualitative components. For example, focus group themes like the insufficiency of training are not sufficiently linked to survey results. It is also suggested that the authors further discuss how the unique challenges of the Philippine healthcare and education systems, such as technological infrastructure or ethical training gaps, influence the findings.

Have any limitations of the research been acknowledged?

Partly

Is the work clearly and accurately presented and does it cite the current literature?

Yes

If applicable, is the statistical analysis and its interpretation appropriate?

Partly

Are all the source data underlying the results available to ensure full reproducibility?

Partly

Is the study design appropriate and is the work technically sound?

Partly

Are the conclusions drawn adequately supported by the results?

Partly

Are sufficient details of methods and analysis provided to allow replication by others?

Partly

Reviewer Expertise:

medical humanities, professionalism, globalizaion and cultural studies, curriculum design

I confirm that I have read this submission and believe that I have an appropriate level of expertise to state that I do not consider it to be of an acceptable scientific standard, for reasons outlined above.

# Response

**Published:** 2025-03-22

**Authors:** Robbi Miguel Falcon

**Affiliations:** College of Medicine, University of the Philippines Manila, Manila, Metro Manila, Philippines

We would like to thank the reviewer for taking their time and effort to review the manuscript, once again. We greatly appreciate your valuable suggestions and comments to improve the quality of the manuscript. We explain here how we edited and addressed the concerns and corrections you raised in your review, as well as the additional edits we thought were necessary to improve the paper. The revisions and answers are highlighted in red font in the manuscript.

**Comment:** The research questions are quite broad, attempting to cover attitudes toward AI in education, its application in clinical practice, and ethical concerns. This breadth risks diluting the focus, especially given the small sample size and limited institutional scope.

**Response:** The study aims to provide a preliminary basis for future investigations on the impact of AI on medical education. Rather than limiting its scope to one aspect of the field, it attempts to explore the field as a whole by providing a wide array of directions that future studies can pursue and validate, especially in the Philippine context. Hence, it decided to adopt multiple surveys covering different aspects of medical education in order to better provide a basis for why the field is in need of further investigation.

**Comment:** Questions about AI's impact on specific aspects of medical practice (e.g., diagnostics, doctor-patient interactions) could be better justified with prior literature or hypotheses.

**Response: **Discussion The basis for the inclusion of questions related to medica practice was expounded upon, based on the findings from previous studies. References: [1] Moldt, J. A., Festl-Wietek, T., Madany Mamlouk, A., Nieselt, K., Fuhl, W., & Herrmann-Werner, A. (2023). Chatbots for future docs: exploring medical students’ attitudes and knowledge towards artificial intelligence and medical chatbots. *Medical Education Online*, *28*(1), 2182659. [2] Sun, L., Yin, C., Xu, Q., & Zhao, W. (2023). Artificial intelligence for healthcare and medical education: a systematic review. *American Journal of Translational Research*, *15*(7), 4820.

**Comment: **The paper briefly mentions AI equity and challenges in AI integration in developing countries but needs to deeply contextualize these challenges within the Philippine healthcare and education systems.

**Response: **Introduction Further context on the Philippine healthcare and educational system was elaborated on in the Introduction. This was correlated to concepts in digital health and the possible inequities which may arise due to socioeconomic disparities.

**Comment: **A stronger linkage to specific barriers in the Philippine medical education context (e.g., resource limitations and faculty readiness) would provide a more robust justification. While the study is pioneering in the Philippine context, the research questions are similar to those explored in other global studies. Cultural attitudes toward technology or systemic challenges in medical training can also be provided.

**Response: **Introduction Further justification of the exploration of the role of AI in medical education specifically was expounded upon further in the text. This was related to documented findings in studies done in other countries outside of the Philippine context.

**Comment:** The participants were 20 students, and the sample of 20 students was inadequate for quantitative analysis, especially for generalizing findings—the participants' demographic needs to be provided, such as the learning stage of this cohort.

**Response:** 2.1 Study Design and Participants The learning stage of participants was included in the demographic profile of the study. Furthermore, since the current study employed a mixed-methods approach with the surveys accompanied by focus group discussions with the participants, the need for a large sample size was not deemed necessary for this pilot study. 

 Reference: [1] Hennink, M., & Kaiser, B. N. (2022). Sample sizes for saturation in qualitative research: A systematic review of empirical tests. *Social science & medicine*, *292*, 114523. [2] Vasileiou, K., Barnett, J., Thorpe, S., & Young, T. (2018). Characterising and justifying sample size sufficiency in interview-based studies: systematic analysis of qualitative health research over a 15-year period. *BMC medical research methodology*, *18*, 1-18.

**Comment:** The study did not provide more details about the validation of the questionnaire.

**Response: **2.3 Questionnaire The basis for validity was included in the text.

**Comment: **The study relies solely on descriptive statistics without exploring relationships or differences using inferential statistics. This limits deeper insights into the data, such as identifying factors influencing attitudes. There is no comparison with other cohorts, such as students from different institutions or countries, which would help contextualize the findings and highlight unique aspects of the Filipino medical education system.

**Response: **Discussion   Limitations Since this is the first pilot study done in the Philippine context, subgroup analyses were not performed. However, discussions comparing the contexts of Filipino medical students to medical students from other institutions were elaborated on.

**Comment: **The focus group questions are not directly mapped to specific quantitative survey questions or subsections. This makes it harder to draw precise links between the datasets. How the qualitative data was triangulated with the quantitative one needs to be elaborated. In other words, how the qualitative data complemented the quantitative data requires further explanation.

**Response:** 2.4 Focus Group Discussion Integration of the quantitative and qualitative sections of the study was done following Guetterman (2017), which involved generation of meta-inferences based on the findings from each methodology used, and legitimation of the findings based on previous works.   Reference: [1] Guetterman, T. C. (2017). Designing a rigorous mixed methods research study. *질적연구*, *18*(1), 1-16.

**Comment: **While ethical concerns are a prominent theme in the survey, they need to be sufficiently emphasized in the focus group questions. For example, data privacy and liability concerns mentioned in the quantitative section could have been probed further in the qualitative discussions.

**Response:** 3.1.5 Ethical Considerations for AI Use in Medicine This comment is duly noted and expounded on further in future iterations of the study. For the purpose of this pilot investigation however, the scope will be retained to the initial answers provided by the participants.

**Comment:** The analysis of qualitative data (interviews) needs to be more clarified. For instance, did the authors use thematic analysis?

**Response: ** 2.4 Focus Group Discussion The authors utilized an inductive content analysis to analyze transcripts of the focus group discussions conducted. Codes generated served as the basis for the meta-inferences formed from the mixed-methods approach employed.   References: [1] Byrne, D. (2022). A worked example of Braun and Clarke’s approach to reflexive thematic analysis. *Quality & quantity*, *56*(3), 1391-1412. [2] Braun, V., & Clarke, V. (2023). Toward good practice in thematic analysis: Avoiding common problems and be (com) ing a knowing researcher. *International journal of transgender health*, *24*(1), 1-6.

**Comment: **The discussion section effectively summarizes the study’s key findings and aligns with previous literature. However, while the study uses a mixed-methods approach, the discussion does not fully integrate insights from the quantitative and qualitative components. For example, focus group themes like the insufficiency of training are not sufficiently linked to survey results. It is also suggested that the authors further discuss how the unique challenges of the Philippine healthcare and education systems, such as technological infrastructure or ethical training gaps, influence the findings.

**Response: **3.2 Focus Group Discussion   Discussion The results of both the survey and focus group discussion were more explicitly integrated in the text. An elaborate discussion of the integration, relating to previous studies done in other countries, was also included. This was further supported by studies which highlight the gaps of medical education in the Philippine context, to further emphasize the unique aspects of the medical education ecosystem of the country. Thank you very much!
